# Case report: rare case of mechanical bowel obstruction due to strangulation by gastric stimulator electrodes

**DOI:** 10.1186/s12893-015-0022-4

**Published:** 2015-03-30

**Authors:** Hans Lederhuber, Stephan Axer, Christof Ihle

**Affiliations:** Department of General Surgery, Torsby Hospital, Värmland County Council, Lasarättsvägen 8, 68529 Torsby, Sweden

**Keywords:** Gastroparesis, Ileus, Implantable stimulation electrodes

## Abstract

**Background:**

Implantation of a gastric stimulator is a feasible surgical therapy for patients with therapy refractory gastroparesis. In addition it seems to be a promising alternative for treating morbid obesity. We present for the first time the surgical emergency of small bowel obstruction due to strangulation by gastric stimulator electrodes.

**Case presentation:**

A 59-year-old Caucasian female had undergone implantation of a gastric stimulator to cope with the symptoms of a partial gastroparesis. Eight years after the operation, the patient began to present repeatedly to different hospitals because of abdominal pain and nausea. Symptoms and imaging indicated ileus, which could always be treated conservatively. The underlying pathology could not ultimately be determined and the symptoms were eventually considered gastroparesis-related. After two years the patient was finally referred in circulatory shock due to peritonitis with underlying small bowel obstruction. Emergency laparotomy revealed small bowel strangulation by the gastric stimulator electrodes.

**Conclusion:**

Repeated presentation of a patient with an unfamiliar treatment modality must raise suspicion of unusual complications. Specialist surgeons treating with innovative methods should provide proper information that is accessible to everyone who might have to treat possible complications.

## Background

Implantable gastric stimulation is a method to stimulate the stomach in order to overcome gastroparesis [1-6] and to treat morbid obesity [7-10]. During a laparoscopic intervention two electrodes are placed on the anterior medial wall of the stomach. The distal end of the electrodes is connected with the stimulation device, which is implanted in a subcutaneous pocket in the abdominal wall [4].

Patients with gastroparesis suffer from delayed emptying of the stomach which can lead to varying problems such as nausea, vomiting, abdominal pain and early satiety with concomitant weight loss [11]. Gastroparesis is not caused by mechanical obstruction but by abnormal gastric myoelectric activity or abnormal gastric motility [12]. There is a wide range of pathologies that can lead to gastroparesis. Two well-known culprits are diabetes mellitus [13] and vagotomy (e.g. due to index gastric surgery) [14]. Nonetheless, some cases of gastroparesis have to be classified as idiopathic [15,16].

The traditional treatment of gastroparesis is in most cases symptomatic. Some patients benefit from mere diet modification, others need additional pharmacotherapy [17,18]. However, long-term medication comes with a range of side effects, which can deteriorate patients’ quality of life and therefore is not often tolerated. In addition, there are a number of non-responders [19]. Surgical treatment of gastroparesis can be considered an option when medical treatment has failed. Surgical treatment includes gastrostomy or jejunostomy feeding tubes, gastrectomy and gastric stimulation [20-24]. All surgical treatment options of gastroparesis have in common that evidence from randomized controlled trials for the success of such intervention is lacking [25].

Nowadays, another indication for gastric stimulation is morbid obesity. With obesity being a disease-entity with huge socioeconomic impact [26-28], medical and surgical therapies arose during the past years. However, pharmacotherapy often comes with a range of unacceptable side effects [29,30]. Surgical intervention produces long-term weight loss [31,32] however it’s invasive with respective morbidity and mortality. Moreover, internal herniation after laparoscopic gastric bypass procedures has become a pending problem [33,34] and complications as well as limitations in this older-growing operated population are not yet predictable [35]. Implantation of a gastric stimulator represents a less invasive treatment option for some patients. Although it is approved for clinical use, it is not fully eluted how gastric stimulation leads to weight loss.

## Case presentation

A 59-year-old Caucasian female patient was referred to our emergency department by ambulance from another hospital where no operative service was available at night. On arrival in the emergency department, the patient presented in circulatory shock with pulse around 160 bpm and blood pressure 65/35 mmHg. Her laboratory results were remarkable for: pH acidic at 7.13, pCO_2_ 8.6 kPa, PO_2_ low at 5.3 kPa, lactate high at 9.2 mmol/L, BE −8.8 mmol/L. Her CRP was 48 mg/L and WBC low normal at 3.5*10^9^/L. On examination, she showed all signs of general peritonitis. The patient was prepared for emergency laparotomy immediately.

**Figure 1:**
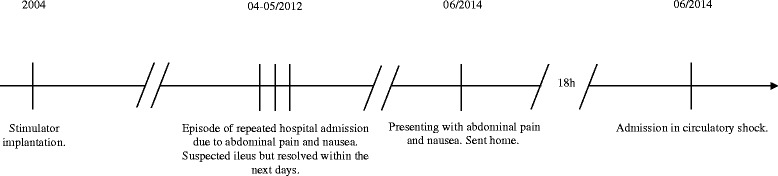
**Timeline summarizing important clinical events.**

According to the patient’s journal records she had undergone laparoscopic implantation of a gastric stimulator in 2004 due to gastroparesis. The patient had a medicated hypertension and a medicated hyperlipidaemia in her medical record but no further conditions. She had earlier been operated with open surgery for appendicitis, C-section and ovarian cysts. Between April 2012 and June 2014 the patient had shown up several times to different hospitals in our county (Figure 1). She had then presented with abdominal pain, bloating and fever. Mechanical small bowel obstruction had been suspected each time but either the CT scans had been inconclusive or a sub-ileus condition had been resolved during small bowel series. Discussion with the respective gastric surgeons had revealed that the patient had a partial gastroparesis due to idiopathic neuropathy. The patient’s symptoms had eventually been interpreted as pseudo-obstruction and colonoscopy had been recommended. This examination however had not shown any pathology besides minor diverticulosis. The patient’s recurring symptoms had got better after initiation of a treatment with neostigmine and erythromycin before meals. Follow-up at a gastrointestinal centre had been planned. June 2014 the patient showed up to her nearest hospital during early morning, once again with abdominal pain and nausea. The abdominal tenderness was focused around the umbilicus but physical examination was otherwise without remark. Blood samples showed CRP at 1 mg/L, WBC at 10.8*10^9^/L. Body temperature was 37.1°C, blood pressure 120/79 mmHg and pulse 51 bpm. The abdominal pain was assessed as functional and the patient was sent home. The same evening however the patient was referred by ambulance to the same hospital. She was tackycardic (around 140 bpm) and low in blood pressure (about 75/50 mmHg). Body temperature was 35.8°C. She had vomited several times before she had ringed the ambulance. Clinically she presented this time with peritonitis, no bowel sounds. After she’d been stabilized initially a CT scan showed signs of small bowel obstruction.

**Figure 2:**
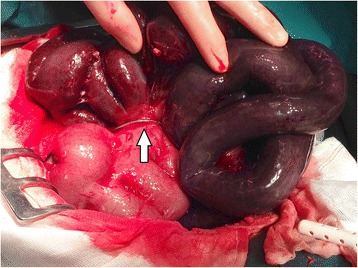
**Stimulation leads strangulating parts of the small bowel.** The white arrow marks the leads.

Our hospital was contacted when it became clear that surgery was inevitable and that there were no resources available at night at the other two nearer hospitals. This was when the patient was referred and presented as described above. After immediate admission to the operating room the abdomen was opened with a midline laparotomy and bloody-serous fluid was found in the abdominal cavity. Approximately two thirds of the small bowel were necrotic. No adherences between the bowel and the abdominal wall could be seen but interenteric adherences were present without causing a stricture. We identified that herniation of small bowel through the loop of the stimulation-leads was causing a mechanical strangulation ileus of the small bowel. To prevent recurring strangulation, it was decided to cut the leads. The strangulated bowel was not recovering and had to be resected. Continuity was provided with a hand-sewn end-to-end enteroentero-anastomosis. The patient was left with 120 cm small bowel (Figure 2). The leads were shortened to the level of the abdominal wall and the stimulation device was left in place. Perioperatively a treatment with cefotaxime and metronidazole was started according to national standards. The patient was extubated the first postoperative day and was able to be discharged from our hospital on the eighth postoperative day.

Two months later the patient was seen in our day-clinic for follow up and for explanting the stimulation device under local anaesthesia. She was feeling well but experiencing some symptoms of her gastroparesis anew.

## Conclusions

Gastroparesis and morbid obesity are two quite different conditions. However, they have both close relations to the gastrointestinal tract. Both conditions can be treated by diet modification or pharmacotherapy. These conditions also have in common that the first and the latter therapeutical approaches use to fail in certain patients for a variety of reasons. This is why surgical strategies exist for gastroparesis as well as morbid obesity.

The backdraft of surgical therapy is that it always comes with morbidity and mortality. The attempt to overcome these limitations can be seen as the incitement to develop new and better surgical techniques and has led to the surgical state of art we see today.

Compared to gastrectomy or Roux-en-Y gastric bypass, the implantation of two electrodes during minimal invasive surgery is by far less invasive. The possibility to merely switch off the stimulation device if needed is even more alluring as gastrectomy is not reversible and reversion of a gastric bypass is challenging. However, as seen in the case presented, not even implantation of a gastric stimulator is totally risk-free. The issue of stimulator placement, lead length and lead placement in particular might be worth to raise if adverse events should occur more frequently.

Presenting this extremely rare case of small bowel strangulation, we want to highlight some major concerns that we think can be transferred to similar scenarios:

Using novel treatment approaches is desirable when it comes with a benefit for the patient but complications after such treatment must be managed professionally. One gets into situations of vague ethical boundaries when this management fails due to lack of knowledge of the new approach. Similar problems were seen some years ago when the first patients presented to emergency departments around the country with internal herniation after gastric bypass surgery. It was widely unknown that such a complication even existed, especially as many patients did not present to a bariatric center but to some hospital nearby with a general surgeon in charge. A general surgeon should not be expected to be capable of solving an internal herniation laparoscopically. But every general surgeon nowadays has to know the possible complications and the workup of a patient who has undergone bariatric surgery and is now presenting with abdominal pain and nausea.

In the case presented the knowledge transfer from the center where the gastric stimulator had been implanted to the patient’s nearest county hospitals had probably not been optimal. Potential pitfalls and especially herniation risk had obviously not been pinpointed clearly enough. And here we address the gist of this case: what might seem obvious for a sub-specialist surgeon who is dealing with a condition on a daily basis might not be obvious at all for a general surgeon who is not even aware of the problem. Thus it would be desirable if sub-specialists who apply techniques that are not common surgical knowledge would provide proper documentation, patient information and above all, knowledge transfer to their colleague surgeons. On the other hand, this case also demonstrates the responsibility of the local general surgeon. Patients with an unfamiliar treatment modality who repeatedly present to the emergency department must raise suspicion. If additional workup is inconclusive, close contact to the sub-specialists seems advisable. Finally it might be recommended to consider falling back upon basic surgical procedures. In the case presented, diagnostic laparoscopy would have been a fast forward procedure with limited risks and high potential to spot the problem.

In conclusion we want to summarize that the description of small-bowel strangulation through leads of a gastric stimulator might be a zebra, but cases like this should raise awareness for the problems that come with the introduction of new surgical techniques.

## Consent

Written informed consent was obtained from the patient for publication of this case report and any accompanying images. A copy of the written consent is available for review by the Editor of this journal.

## Abbreviations

BPM beats: Per minute
CRP: C-reactive protein
CT: Computer tomography
WBC: White blood cell count
